# Local Immune Control of Latent Herpes Simplex Virus Type 1 in Ganglia of Mice and Man

**DOI:** 10.3389/fimmu.2021.723809

**Published:** 2021-09-15

**Authors:** Anthony J. St. Leger, David M. Koelle, Paul R. Kinchington, Georges Michel G. M. Verjans

**Affiliations:** ^1^Department of Ophthalmology and Immunology, University of Pittsburgh School of Medicine, Pittsburgh, PA, United States; ^2^Department of Medicine, University of Washington, Seattle, WA, United States; ^3^Department of Laboratory Medicine and Pathology, University of Washington, Seattle, WA, United States; ^4^Department of Global Health, University of Washington, Seattle, WA, United States; ^5^Vaccine and Infectious Diseases Division, Fred Hutchinson Cancer Research Center, Seattle, WA, United States; ^6^Benaroya Research Institute, Seattle, WA, United States; ^7^Department of Ophthalmology and Molecular Microbiology and Genetics, University of Pittsburgh School of Medicine, Pittsburgh, PA, United States; ^8^HerpesLab.NL, Department of Viroscience, Erasmus Medical Center, Rotterdam, Netherlands

**Keywords:** HSV-1, latency, ganglion, mouse, human, intrinsic immunity, innate immunity, adaptive immunity

## Abstract

Herpes simplex virus type 1 (HSV-1) is a prevalent human pathogen. HSV-1 genomes persist in trigeminal ganglia neuronal nuclei as chromatinized episomes, while epithelial cells are typically killed by lytic infection. Fluctuations in anti-viral responses, broadly defined, may underlay periodic reactivations. The ganglionic immune response to HSV-1 infection includes cell-intrinsic responses in neurons, innate sensing by several cell types, and the infiltration and persistence of antigen-specific T-cells. The mechanisms specifying the contrasting fates of HSV-1 in neurons and epithelial cells may include differential genome silencing and chromatinization, dictated by variation in access of immune modulating viral tegument proteins to the cell body, and protection of neurons by autophagy. Innate responses have the capacity of recruiting additional immune cells and paracrine activity on parenchymal cells, for example *via* chemokines and type I interferons. In both mice and humans, HSV-1-specific CD8 and CD4 T-cells are recruited to ganglia, with mechanistic studies suggesting active roles in immune surveillance and control of reactivation. In this review we focus mainly on HSV-1 and the TG, comparing and contrasting where possible observational, interventional, and *in vitro* studies between humans and animal hosts.

## Introduction

Herpes simplex virus type 1 (HSV-1), a human neurotropic alphaherpesvirus, is a prevalent human pathogen with a global cumulative burden of 3.7 billion infections (1). HSV-1 incidence, functionally equivalent to seroconversion and primary infection, peaks in early and mid-childhood in westernized societies. In a US cross-sectional study repeated every 10 years, prevalence in adults has gradually declined since 1999 (2). Worldwide seroprevalence has been reviewed and tends to be higher, up to 87%, in lower and middle income countries, and overall slightly higher in females (1). Adult primary HSV-1 infection is recognized in the sexual transmitted infection literature in more than half of clinical first episodes of genital herpes in the US and areas of Europe (3). This is related, likely causally, to decreasing childhood prevalence with more persons initiating sexual activity when seronegative.

HSV-1 enters the body by infecting the epithelial layer of mucosal surfaces and skin (4). After initial infection and replication, during which the viral genes are expressed in a coordinated fashion resulting in new progeny virus that facilitate dissemination to surrounding cells or infect new individuals, HSV-1 gains access to the termini of local sensory neurons. Retrograde axonal transport carries the virus to the neuronal cell bodies in sensory ganglia, most commonly the trigeminal ganglion (TG) when persons are infected *via* the orofacial and ocular route, where subsequent lifelong latency is established. Latently HSV-1-infected neurons harbor the non-productive viral genome as histone-associated circular episomal structures that are largely transcriptionally silent except for a family of non-coding latency-associated transcripts (LATs) (4). However, not all latently-infected HSV-1 neurons express LAT and some show rare and sporadic lytic gene transcription and protein expression, suggesting that latency is a dynamic state, and may reflect heterogeneous neuron populations harboring the HSV-1 genome. Poorly defined stimuli can cause the virus to reactivate periodically and travel *via* anterograde axonal transport to the periphery to cause recurrent HSV-1 infections and disease ranging from clinical to asymptomatic. Studies on HSV-1 infected rat neuron cultures have suggested that reactivating genomes transcribe lytic genes in two phases during reactivation (5). The first stage is characterized by a transient burst of lytic gene transcription outside of the established lytic cascade and independent of the viral tegument protein VP16. A similar synchronous wave of broad gene expression has been observed following *ex vivo* reactivation in which latently HSV-1-infected mouse TG explants were combined with nerve growth factor deprivation (6). The second phase of reactivation mimics lytic infection, which commences when threshold amounts of key viral proteins are synthesized during phase I (including the IE gene activator VP16) leading to amplification of viral DNA and production of infectious HSV-1 (5). Asymptomatic shedding of virus can be frequent: for example, amongst 102 immunocompetent adults with chronic HSV-1 infection swab-sampled for a median of 60 consecutive days, viral DNA was present in 9% of swabs overall. For individual persons, rates of HSV-1 shedding ranged up to 47% of days (7). These data further highlight differences for HSV-1 between animals and humans and must reflect, at some level, ganglionic control.

Given the high incidence and permanence of HSV-1 infection, the health care burden of HSV-1 is considerable worldwide. Severe syndromes are associated with recurrence and ganglionic reactivation, including the sight-threatening HSV stromal keratitis (HSK), acute retinal necrosis, and life-threatening HSV encephalitis (HSE) in adults. Neonatal HSV is also sometimes due to HSV-1 but with lower burden than HSV-2 (8) and has recently been associated with maternal primary infection and lack of transplacental antibody (8–10). Other severe manifestations that are less likely to reflect ganglion biology include childhood HSE and HSV hepatitis in the immunocompetent person, usually related to primary infection and/or defective innate immunity (11, 12) In immune suppressed persons, recurrent infections can be prolonged, severe, or systemic from epithelial to visceral sites. Host adaptation by HSV-1 is profound and leads to differences in clinical syndromes between humans and model organisms, albeit the host and viral genetic basis is poorly understood. Unlike humans, HSV in the murine model does not show evidence of spontaneous recurrence. Hyperthermic or containment stress (13, 14), drugs that modulate histone modifications (15), or corticosteroids (16) can trigger ganglionic molecular evidence of reactivation *in vitro* or *in vivo*. There appear to be further host restriction points, such that recovery of infectious virus from peripheral in innervated sites is very uncommon. Guinea pigs infected genitally with HSV-1 can have sporadic clinical and virologic recurrence after clearance of the inoculum that are thought associated with ganglionic reactivation (17). This syndrome resolves over time. Rabbits are prone to spontaneous HSV-1 recurrences in some ocular models (18) and have been used for molecular virology, vaccine and drug treatment studies (19, 20). HLA transgenic animals are available and have been used in interventional studies of immune checkpoint blockage (21). Recurrent clinical syndromes are not noted in old-world non-human primates (22), while some new world primates are hypersusceptible to fatal HSV-2 primary infection and are not well studied for HSV-1 (23).

Control of HSV-1 acute infection, latency and reactivation in the ganglia occurs at many levels ranging from epigenetic modifications of histones near critical viral gene promoters to infiltration and retention of virus-specific T-cells. The scope of viral immunology has extended recently from specific T and B cells with hypervariable receptors to leukocyte and cell-intrinsic immunity. In our review we discuss studies on intrinsic, innate and adaptive immunity that are potentially involved in the control of neuronal HSV latency in ganglia. Our intention is to spark discussion and collective research initiatives aimed to fully identify ganglionic cell types and define targets for investigative and ultimately therapeutic manipulation. Recommendations are made to facilitate convergence towards *in vitro* and *in vivo* model systems and defined reagents with the goal of facilitating inter-laboratory interpretation of research findings and ultimately progress in reducing the clinical consequences of latent ganglionic HSV-1 infection.

## Intrinsic Immunity

Mammalian immune systems use three interacting mechanisms to eliminate infections and maintain homeostasis. Innate immunity is mediated by germ-line encoded pattern recognition receptors (PRR) and toll-like receptors (TLRs) that recognize specific pathogen-associated molecular patterns (PAMPs). Detection of HSV-1 by leukocytes such as plasmacytoid dendritic cells with secretion of IFN-α (24, 25) to stimulate interferon-stimulated genes (ISG) is exemplary of innate immunity as typically defined (26). There is debate about whether the IFNs/ISG pattern in HSV-infected epithelial tissues reflects type 1 (α and β) or II (γ IFNs (25, 27); the latter is a classic cytokine from innate lymphocytes such as NK and TCRγδ cells as well as specific T-cells. Intrinsic immunity overlaps with innate immunity and also consists of constitutively-expressed germline encoded restriction factors that provide an immediate antiviral response, as well as physical barriers, but generally lacks cell to cell communication and thus response amplification. Adaptive immunity is mediated by antigen-specific receptors generated by rearrangement of genes of the B- and T-cell receptor loci. In comparison to innate and adaptive immunity, the role of intrinsic immunity in counteracting HSV-1 infections, especially latency, is less known but is becoming a subject of some interest. Effector mechanisms of intrinsic immunity to HSV-1 infection include inhibition of specific stages of the replication cycle (e.g., nuclear restriction factors and RNA interference) and induction of degenerative processes (e.g., autophagy, especially in neurons (28). Intrinsic immunity appears important to control HSV-1 infection and directing the fate of the genome into the latent state, particularly in the absence of lytic proteins entering the cell as the virion or those viral proteins first expressed upon viral DNA delivery.

### Nuclear Restriction Factors and the Lytic/Latent Decision Process

The first aspect of intrinsic immunity is the rapid association of the capsid-released HSV DNA genome with host restrictive factors. Virion DNA is not histone-associated, but within an hour in epithelial cell nuclei, [and longer in neurons (29)], the HSV genome co-localizes with Pro Myelocytic Leukemia (PML) proteins, components of the nuclear domain 10 (ND10) complex and components of small ubiquitin like modification (SUMO) complex. The genome can then develop widespread repressive marks of facultative chromatinization (30–33). The eventual fate of the default chromatinized viral genome differs for epithelial cells and neurons. In neurons, more repressive markers of heterochromatin can develop, correlating with silencing of most gene expression (with the exception of the LAT locus), while in most epithelial cells, more permissive euchromatin states develop, with an eventual loss of histones across the viral genome. This is partly a result of activities encoded by tegument and newly expressed immediate early (IE) proteins that drive a lytic infection, and their differential delivery/expression in the two cell types. In epithelial cells, pro-lytic proteins of the virion tegument adapt the host cell to infection, override many intrinsic activities and stimulate expression of specific IE proteins, particularly ICP0. For example, virion tegument proteins counteract host PRR and innate signaling mediators to evade DNA sensing, while others facilitate IE gene transcription. HSV-1 tegument proteins VP11/12 (34) and VP22 (35), and the IE expressed protein ICP27 (36), counteract the host protein STING (stimulator of interferon genes, a cytosolic DNA sensor/signal mediator) and its signaling though TBK1 (TANK-binding kinase 1) and IRF3 (interferon regulatory factor 3) in the early innate immune response to infection. The tegument protein VP16 recruits the cytoplasmic host cell factor 1 (HCF-1) to the nucleus and brings the transcription factor Oct-1 and chromatin demethylases to IE gene promoters to promote their transcription [see review in (37)]. As succinctly put by Sawtell et al., VP16 sets the lytic/latent balance of HSV infection in the TG (38). VP16 also has suggested roles in reactivation and the transition from Phase I to Phase II reactivation (5). A poor reactivation phenotype of HSV-1 seen in rodent models of latency and reactivation has been proposed to be a consequence of is species-specific differences in Oct-1 that affects VP16-mediated activation of IE gene promoters (5). VP16 also promotes euchromatin states by recruiting the lysine-specific histone demethylase 1A (LSD1) to demethylate the repressive epigenetically modified DNA histones H3 (H3K9me2 and H3K9me3); theJMJD2 histone demethylases acting on H3K9me3; and the H3K4 methyl transferases (39–41). These associations have been proposed as a potential target for anti-HSV therapies (42). VP16 has additional anti-innate activities that block NFkB activation and the upregulation of IFN-β, by binding the co-activator of IRF3 (43).

Upon VP16 induction of IE gene expression, the central role of IE proteins emerges, particularly ICP0. This pro-lytic HSV-1 protein has E3 ubiquitin ligase activities that target many intrinsic and innate antiviral proteins in the nucleus and ND10 domains, including PML and the key HSV DNA sensor IFI16 (44–47). ICP0 also promotes histone removal, the acetylation of histones on the genome and in general, other chromatin modifying processes that strongly favor the lytic infectious process. ICP0 also sequesters the interferon regulatory factor IRF3 (48, 49). As such, *the absence* of ICP0 in neurons favors the repressive chromatin state driven by both intrinsic and innate responses that are not counteracted.

The physical mode of axonal infection in neurons also favors the default outcome to repressive heterochromatin and gene silencing. HSV-1 infections of neurons usually initiate at distal axon sites within the skin or mucosa. Upon entry, many outer tegument proteins (including VP22, VP11/12, and VP16 for example) dissociate from the capsid. These are then poorly delivered to the neuronal nuclei in ganglia. This could results in inefficient counteraction of intrinsic and innate PRR and signaling mediators, thus favoring establishment of latency (50). For example STING would hereby not be counteracted by VP11/12 or VP22 (34). STING is a particularly important mediator in neurons, as HSV-1-infected STING knock-out mice die rapidly due to uncontrolled virus replication, spread to the CNS and encephalitis (51–53).

It is important to note that the intrinsic default repression of the HSV genome in neurons has to be a reversible process, at least in a fraction of neurons or neuronal subtypes. This permits HSV reactivation following the poorly defined but multiple stimuli such as stress, altered neuronal hyperexcitability states, or as recently shown, in response to cytokines such as IL-1 (54).

### Promyelocytic Leukemia Protein and the Nuclear Domain 10 Complex

HSV-1 genome in neurons rapidly co-localizes with PML, a member of the Tripartite Ring Interaction Motif protein group, also called TRIM19 (31). PML is a major component of ND10 repositories that encages intrinsic chromatin remodeling proteins that include sp100, the death domain associated protein hDAXX and alpha-thalassemia/mental retardation syndrome X-linked protein ATRX. Both hDAXX and ATRX are recruited independently of PML to the genome and promote the maintenance of chromatin and viral gene repression (55, 56), since depletion of any of these factors results in greatly increased infectivity of HSV-1 lacking ICP0 (57, 58). Viral DNA and PML co-localize rapidly in cultured neurons of both murine and human origin (30, 31, 59, 60), forming structures that have been called viral DNA containing PML-nuclear bodies (vDCP-NB) that are also DAXX/ATRX/SP100-positive in the absence of ICP0 expression. Neurons have PML-ND10 complexes, but recent work suggests ND10 form more cohesively when neurons are exposed to IFN-I, such as might occur when axons in peripherally infected tissues are exposed and there is subsequent signaling along the axon (61). The silenced viral genomes subsequently become enriched with characteristic heterochromatic histone modifications, such as histone H3 di- and tri-methylated at lysine 9 (H3K9me2/3) and H3K27me3 (29, 62–64). These changes follow PML association and the recruitment of chromatin remodeling components, such as the HIRA complex of histone H3.3 and its chaperone factors UBN-1, CABIN1 and ASFa1 (32, 33). This occurs in epithelial cells infected at low multiplicity of infection that do not express ICP0 (30, 31). Histone H3.3 becomes lysine 9 trimethylated at multiple sites of the genome, correlating with full silencing of HSV-1 gene expression (31, 32). Importantly, similar structures to the vDCP-NB have been seen in both murine and human latently HSV-1-infected TG neurons (31, 59, 65). A new member of the TRIM protein family, TRIM22 was recently identified as also having a role in the intrinsic response to HSV (66). TRIM22 was a restriction factor against HSV-1 that promotes chromatic compaction of viral DNA at the IE gene loci, hereby inhibiting virus yields through epigenetic silencing. Unlike PML/TRIM19, TRIM22 was not degraded by ICP0 as a result of ubiquitination, but was still counteracted by this IE protein. It remains to be seen if this has roles in neurons to play a role during the pressure to establish latency.

### Gamma-Interferon-Inducible Protein 16

IFNγ-induced protein 16 is one of the key DNA sensors for HSV and is strongly counteracted through ubiquitination and proteosomal degradation mediated by ICP0. The rapid association of the HSV genome with PML is independent of IFI16 and IFN-stimulated gene induction (33), but the DNA sensors IFI16, cGAS and STING are apparently recruited to the same complex in the absence of ICP0. The DNA sensors cGAS and IFI16 would be active in neurons where tegument protein counteractors such as VP22 do not reach the neuron nucleus (35). Absence of VP16 delivery results in inefficient expression of ICP0 and its central role in overriding intrinsic activities of proteins associated with ND10 domains and IFI16 (60, 65). IFI16 is also depleted in an ICP0-independent manner involving the VHS outer tegument protein [which would also remain at the infecting axon site (67); reviewed in (68)]. These conditions suggest IFI16 is active in infected neurons and would stimulate the innate inflammasome-signaling responses and IFN response. That IFI16 is counteracted in two mechanisms, by both a tegument protein (VHS) and ICP0, underscore its importance as an HSV DNA sensor that could influence lytic latent decisions (67–69). IFI16 contributes to the intrinsic response by recruiting histone modification enzymes on heterochromatin, particularly H3K9 marks (70, 71).

### Small Ubiquitin-Like Modifier

Newly entering viral DNA also recruits core components of the SUMOylating pathway, independently of PML (72). SUMO modifications act through interactions with SUMO interaction motifs (SIM) and in part, modify proteins of the ND10 complex and their interactions including PML in HSV infected cells. SUMO is known to regulate and modify numerous cellular processes including transcription, the stress response and the cell cycle as well as several aspects of immunity [reviewed in (73)]. SUMO-signaling is a component of repression, since depletion of the ubc9 ligase component of the SUMO complex results in increased permissiveness to HSV-1 infection (74). SUMO modification, SUMO-SIM interactions, and a functionally active SUMO pathway are all thought to contribute to the recruitment of PML-NB-associated restriction factors to viral genomes (72). The protein inhibitor of activated STAT4 (PIAS4) was recently shown to be an important intrinsic antiviral factor recruited independent of PML, and siRNA depletion studies of PIAS4 permitted a more permissive environment for HSV-1 lacking ICP0 (75, 76). A second SUMO protein in the vDCP-NB complex is SUMO ligase protein inhibitor of activated STAT1 (PIAS1) (76). The protein co-operatively contributes to repression of incoming HSV-1 genomes independently of PML, but was enhanced by PML binding and showed additive activity to PML-repressing activities.

### LAT and RNA Interference

The intrinsic repression of the genome by chromatin in neurons is also influenced by both cellular and viral RNAi activities, although the mechanisms are not yet clear. More than 21 viral miRNAs have been identified, although recent studies have suggested that in lytic infections, miRNA biogenesis and nuclear export are inhibited though ICP27 (77). This IE protein is not present during latency, suggesting that host and viral miRNAs retain function. There is great interest in the 8-10 miRNAs made from the LAT primary 8.3 kb transcript (78, 79), the only locus highly expressed in murine and human latently HSV-infected neurons. LAT expression is mediated in part by local chromatin insulation/protection markers and neuronal specific promoter elements (80–83). LAT products have many implied roles aiding in genome repression, the establishment of latency, maintenance of latent genomes by enhancing neuronal survival, and in facilitating efficient reactivation, but the mechanisms are not yet clear and LAT is not essential for any of these processes (see reviews in (84, 85). LAT-encoded miRNAs can regulate expression of key HSV IE protein ICP0 through miR-H2 (62, 63, 86–88). Viral miRNA generated modifications may not only permit long-term latency, but also prime the genome repression for reversal and virus reactivation following the appropriate stimuli. The roles of other miRNA species from the LAT locus include altering other IE gene expression (78, 79), modulating chromatin, antagonizing host intrinsic factors such as ATRX (89) and affecting components of innate immunity. Recent work indicates, for example, miR-H1 and miR-H6 influence efficient reactivation from latency (90), while miR-H8 deletion has little apparent influence in *in vitro* or *in vivo* models of latency (91). The regulation of ICP0 gene expression by miR-H2 influences neurovirulence, but its role of activity in ganglionic models affecting ICP0 expression is less clear (88). Similar analyses are likely underway for all of the HSV miRNAs, so their roles will likely emerge in the near future.

The host expresses miRNA, although HSV lytic infections deregulate miRNA expression (77, 92). Some host non-coding RNAs contribute to the intrinsic and innate responses, including miRNA and circular RNAs (84, 93). Host miRNA are known to influence HSV-1 infection by modulating innate immunity or the IFN-I induced response (94–98). It remains to be determined if any have neuronal-specific processes affecting latency and intrinsic immunity to HSV. Recent work has suggested that mutations in SNORA31 (Small Nucleolar RNA, H/ACA Box 31) can impair intrinsic immunity to HSV-1 in neurons of the central nervous system that may underlie susceptibility to HSV-1 encephalitis (99). This class of small RNA molecules primarily guide chemical modifications of other RNAs, mainly ribosomal RNAs, transfer RNAs and small nuclear RNAs. SNORAR31 is predicted to direct the isomerization of uridine residues to pseudo-uridine in snRNA and rRNA. SNORNA31 appears to act by mechanisms different from TLR3 on HSV-1, since exogenous IFN-I cannot override the heightened HSV-1 infection levels in neurons deleted for SNORA31.

### Autophagy (and Phagocytosis)

This intrinsic immunity mechanism was classically shown to be important for HSV-1 by lesioning the HSV-1 ICP34.5 protein: mutants showed extensive attenuation and greatly increased loss of neurovirulence in mice models of HSV-1 infection and an increased autophagy response. Autophagy is an active and important process in post-mitotic terminal neurons that cannot deplete toxic protein aggregates by cell division (100–103). Indeed, the deletion of proteins critical to autophagic processes in neurons results in progressive neurodegeneration. It has been suggested that autophagy, or rather xenophagy of HSV-1, is perhaps more important than IFN-I responses as a non-destructive mechanism of intrinsic antiviral protection, particularly in the brain (103). Autophagy is also upregulated in response to IFN expression and signaling.

## Innate Immunity

Innate immunity is the first amplifiable line of defense against HSV-1 infection, and is largely a response initiated by PRR signaling following detection of PAMPs (104). There are several membrane-bound and cytoplasmic PRRs that respond to different molecular patterns, with some being more important than others for HSV-1. For example, TLR2 recognizes HSV-1 protein gH/gL and gB, TLR3 recognizes dsRNA intermediates, and TLR9, IFI16 and cyclic GMP-AMP synthase (cGAS) recognize viral genomic DNA respectively (105–107). Downstream activities of PRR signaling lead to production of type 1 and possibly type III IFN (interferon lambda) expression, JAK-STAT mediated induction of the expression of IFN- responsive genes and expression of pro-inflammatory cytokines including interleukin 1β (IL-1β), tumor necrosis factor (TNF) and IL-12 (26, 108). Uninfected cells in close vicinity of virus-infected cells are the main source of these cytokines. Besides their direct antiviral and pro-inflammatory activities, these cytokines modulate long-term antigen-specific adaptive immune responses. The importance of PRR-orchestrated innate immunity is supported by the fact that HSV-1 has developed many countermeasures to escape PPR detection, including interactions between viral proteins and molecules in the host sensor pathways that lead to PRR downregulation (e.g. HSV-1 VHS inhibits cGAS and IFI16 expression) or enzymatic activity disruption (e.g. HSV-1 VP22 inhibits cGAS enzymatic activity and signaling *via* STING) (35, 109). As mentioned regarding intrinsic defense, since neuron axonal infections do not efficiently deliver anti-PRR tegument proteins to the neuronal somata, these PRRs responses can still react. Some important PRR such as TLR3 and their downstream signaling mediators (TRIF, TRAF3 and TBK1) have been identified as a result of genetic studies, where mutations predispose individuals to high incidence of HSE after primary HSV-1 infection without clinical HSV-1 dissemination and other viral infections. These studies suggest that TLR3 has a unique activity to control HSV infection in the brain, but the incomplete penetrance of clinical immunodeficiency in the setting of TLR3-pathway mutations remains to be explained (110, 111). The functional redundancy of PRRs sensing HSV is striking. While potentially the result of a host response to counteract escape mechanisms of HSV during long-term co-evolution, recent studies suggest that PRRs may have unique roles in antagonizing HSV-1 infection in a temporal or even cell/tissue-specific manner. For example, compared to astrocytes and neural stem cells, neurons and oligodendrocytes differentiated from induced pluripotent stem cells (iPSCs) from dermal fibroblasts of TLR3-deficient individuals were selectively impaired in IFN-α/β production and more susceptible to HSV-1 infection (112).

The expression of cytokines also recruits innate immune cells to the site of HSV-1 infection, including neutrophils, macrophages, dendritic cells and natural killer (NK) cells (113, 114). NK cells have emerged as particularly important in control of HSV-1 because severe HSV infections occur in patients with congenital NK deficiencies (115) and NK-depleted mice die rapidly upon corneal HSV-1 infection due to encephalitis (115). Because experimental HSV-1 mouse models are well established to mimic some immunological and pathogenic features of HSV-1 infection in humans, the roles of many individual PRR have been examined by infecting PRR knock-out mouse strains or depleting key cytokines, and this has revealed pivotal roles of PRR signaling and innate immune cells in controlling productive HSV-1 infections (104, 113, 114). This has been studied extensively in the HSV-1 keratitis mouse model, which mirrors the pathogenesis of HSK, one of the most common types of infectious corneal blindness worldwide (116). While reactivations may lead to recurrent corneal HSV-1 infection in humans that is commonly seen as asymptomatic shedding or transient corneal epithelial lesions, it can trigger more severe and potentially blinding corneal disease and the development of immune mediated HSK in some individuals. This can require prolonged and combined antiviral and immunosuppressive treatments that are not always successful (117). Studies in mice have shown that the interplay between local innate and adaptive immune responses, especially CD4 T-cells, determines the outcome of corneal HSV-1 infection in mice. Innate immune cells also exert a dual role, which is exemplified by the role of myeloid-derived cells, potentially neutrophils in HSV-1 keratitis (118). While these cells seem protective in the acute anti-viral response clearing corneal HSV-1 infection, corneal T-cell infiltration and activation leads to a second influx of myeloid-derived cells that are potentially involved in destruction of corneal tissue and laying down less organized (and more opaque) scar tissue in the chronic phase of the disease (119, 120).

Innate immune cells also infiltrate the ganglia during the early phase of local infection, of which the TG has been the most well studied in the case of HSV-1 inoculation of mice *via* the nose or cornea. The infiltration of macrophages, γδT-cells and NK cells and their secretion of cytokines including IL-1, IFN-α/β and IFNγ contribute to impede local HSV-1 replication (121–123). The subsequent influx of macrophages, NK cells and at later stages virus-specific T-cells, coincides temporally with the onset of HSV-1 latency and as such, may be casually associated with termination of productive infection in ganglia (100, 121). In comparison, the role of the innate immune system controlling latent HSV-1 in ganglia is less well defined. This is partly due to the finding that mice deficient in specific innate receptors, cell types and cytokines commonly die due to encephalitis when HSV-1 spreads to the brain before true HSV-1 latency is established (124). However, with a carefully controlled inoculum and assistance of antiviral drugs such as acyclovir, latency can be established in ganglia of these mouse strains (125). Studies using conditional knock-out mice targeting PRRs or other innate immunity factors are warranted for detailed studies.

The innate infiltrate developing in latently HSV-1-infected mouse TG is mostly comprised of mononuclear cells and macrophages (121–123, 126). Studies have revealed that the reactivation of HSV-1 from latently HSV-1-infected mouse TG explant cultures (an *in vitro* model to study immune control of latency as *in vivo* HSV-1 reactivation in mice) have suggested that maintenance of ganglion associated virus-specific CD8 T-cells, discussed in more detail below, are instrumental in preventing HSV-1 reactivation. These act by means of IFNγ, granzyme B (grzB) and perforin mediated transfer of lytic granules in a non-cytolytic manner (127, 128). The role of both CD4 T-cells and macrophages in the ganglia still remains unclear. Contrasting, Ghiasi and colleagues propose that not CD8 T-cells, but CD8α^+^ DC play a more crucial role in maintaining HSV-1 latency (129). Moreover, they suggested that DC induction of T-cell exhaustion to impede the anti-viral effect of CD8 T-cells. Consensus on this topic is needed. In latently HSV-1-infected human TG, the main part of the leukocyte infiltrate also consists of αβ T-cells and some macrophages (130, 131).

Ganglia also contain a resident innate immune cell type, referred to as satellite glial cells (SGC), which completely enwrap neuronal cell bodies (3-10 SGCs/neuron) to form a blood-nervous tissue barrier unique to the peripheral nervous system (PNS) (132). The neuron-SGC interface is complex and involves multiple membrane interdigitations, resulting in a large surface contact area and control of migrating substances to and from the neuron. Besides their supportive role in neuronal homeostasis, SGC provide a physical barrier between neuronal somata and infiltrating immune cells especially cytotoxic T-cells (132, 133). Notably, SGCs closely resemble microglia, sharing phenotypic and functional features with both macrophages and immature DC including phagocytosis, functional PRR expression and upon activation secrete inflammatory mediators such as prostaglandins, IL‐6 and TNF‐α (132–134). Current data suggest that SGCs respond to HSV-1 infection of neurons by secreting IFN-α/β and proinflammatory cytokines (135–137), but also may act as ganglion-resident antigen presenting cells that control local T-cell responses to protect the irreplaceable neuronal somata (132, 138). These effector functions, which have been alternatively assigned to infiltrating DCs and potentially the aforementioned CD8α^+^ DC by other groups (129, 139, 140), may in fact be orchestrated by SGC. Compared to classical innate immune cells, which evidently are essential in clearing productive HSV-1 infection in both the periphery and ganglia, the phenotype and function of SGC hint at their pivotal role in the control of latent HSV-1 infection in ganglia (132, 138). Future studies are warranted to test this hypothesis.

## Adaptive Immunity

Several experimental HSV-1 rodent models, especially the HSK mouse model, has provided detailed insight into the role of T-cells in primary corneal infection and their role in controlling HSV-1 latency in TG. Several days after primary infection at the mouse cornea, adaptive immune cells, mainly T-cells, infiltrate the innervating TG. Their activation status appears a prerequisite to enter the infected TG, resulting in leading to the infiltration of not only HSV-1-specific T-cells but also T-cells of other specificities (141). The specificity of these T-cells and their function in controlling lytic HSV-1 infection at these early times remains unknown. By 1 week post-infection, TG-infiltrating T-cells express activation markers, CD69 and CD27. Additionally, they downregulate CD62L, which is known to be expressed on antigenically naïve cells. Further, virus-specific T-cells may use the chemokine receptors CXCR3 and CCR5 to facilitate migration into the TG, where ligands for these receptors are expressed (141, 142). Genetic deletion of ligands for CXCR3—CXCL9 and CXCL10—in mice results in a reduced migration of CD8 T-cells into the infected ganglion. Intriguingly in humans, HSV-specific memory CD8 T-cells overexpress the chemokine receptor, CXCR3, and the mRNA for ligands of CXCR3—CXCL9, CXCL10, and CXCL11—are increased in HSV pathogenesis (143, 144). As the infection progresses into latency, only virus-specific T-cells are retained in the TG. Within the TG, these cells appear to be true tissue-resident memory (T_RM_) as they express the canonical T_RM_ marker CD69 (131). This pattern of expression is also observed in the mouse skin model of infection (21). Furthermore, these cells position themselves in close proximity to infected neurons and have been found to actively prevent viral reactivation from latency (131, 145). This combined with the analogous positioning of T-cell clusters comprised of CD4 and CD8 T-cells, around TG neurons, underlie the close resemblance in T-cell infiltrates between experimental HSV-1 mouse models and naturally infected human TG.

Virus-specific T-cells are primed in the draining lymph nodes by migratory dendritic cells that migrate from the primary site of infection, either the skin or cornea (146). Conventionally, C57BL/6 mice have been used to study the CD8 T-cell response, since they efficiently prevent HSV-1 from reactivating and express the well-defined major histocompatibility class I complexes (MHC-I) K^b^ and D^b^, which present antigen to CD8 T-cells. MHC molecules in other strains of mice are less well-characterized in HSV infection; however, there has been some identification of HSV-2 CD8 T-cell epitopes in the Balb/c model of infection (147). In the mouse model of corneal HSV-1 infection, it is evident that the CD8 T-cell response in latently HSV-1-infected TG is mainly directed against a single epitope on the HSV-1 protein, glycoprotein B (gB_498-505_). Furthermore, the remaining virus-specific T-cell repertoire is directed against 17 other viral epitopes that are processed from select viral proteins (148). In certain situations when the virus disseminates to the brain, CD8 T-cells also migrate and localize to HSV-infected neurons within the mouse brain (149, 150). An interesting difference between the well-characterized skin model and cornea model of HSV-1 infection is that T_RM_ CD8 T-cells only appear to infiltrate the TG — the site of latent virus —when the cornea is infected, with minimal CD8 T-cell infiltration of the cornea (151). Conversely, T_RM_ CD8 T-cells infiltrate the site of infection in the skin and the site of latent virus in the dorsal root ganglion (DRG) (127, 152). It is currently not clear why higher numbers of HSV-specific CD8 T-cells are not maintained in the cornea as seen in the skin. A possible reason is that the cornea employs several mechanisms that limit excessive inflammation, which may translate into a reduced retention of CD8 T-cells in this tissue (153). In human latently HSV-1-infected TG, HSV-1 proteome-wide scans showed that the local virus-specific T-cell response includes both CD4 and CD8 T-cells that recognize antigenic peptides on one to three HSV-1 proteins, which belong to different kinetic and functional classes of viral proteins. Notably, ICP6 and VP16 were prominent T-cell targets for CD8 and CD4 T-cell subsets in multiple individuals (138). These data imply that full HSV-1 reactivation is not a prerequisite to retain virus-specific T-cells in ganglia with diverse viral protein reactivity. The combined human and mouse data argue that this process involves recognition of the T-cells’ cognate viral antigens produced locally in HSV-1 latently infected ganglia. This strengthened the notion that HSV-1 latency is ‘leaky’, where HSV-1 in some individual neurons may temporarily express lytic proteins potentially representing the first phase of reactivation that is subsequently efficiently controlled by local immune responses from progress to full reactivation (154). Therefore, one could conclude that despite the lack of sensitivity of PCR-based and other more conventional assays, T-cell activation may be a more sensitive measure of viral gene expression during HSV-1 latency.

Understanding the nuances behind the generation of a broad HSV-1 specific CD8 T-cell repertoire continues to be a goal of many. Specifically in C57BL/6 mice, gB_498-505-_specific CD8 T-cells, which account for over 50% of the TG-resident CD8 T-cell infiltrate, remain highly functional. Conversely, the rest of the CD8 T-cell repertoire, loses functionality over time (141, 148). The prevailing hypothesis for this phenomenon was that gB_498-505_-specific CD8 T-cells encountered a “sweet spot” of stimulation that induced an immune response but did not result in overstimulation and eventual loss of function: exhaustion. This hypothesis may not be supported as a recent study showed that placing the expression of gB_498-505_ under the control of different viral promoters alters priming of CD8 T-cells but does not alter frequency or functionality of TG-resident CD8 T-cells during latency (126). The other CD8 T-cells were presumed to be stimulated more robustly during latency, which led to overstimulation and functional exhaustion (155–157). Recent data also suggest that this hypothesis may not be true. First, in the absence of the immunodominant epitope, gB_498-505_, and the associated CD8 T-cell response, the other HSV-1 specific CD8 T-cells better maintain functionality throughout latency (158). Moreover, another study revealed that during priming of the immune response, gB_498-505_-specific CD8 T-cells appear to be programmed in the draining lymph nodes differently compared to the other virus-specific CD8 T-cells, and it is possible that this differential stimulation during early exposure to HSV-1 may dictate future functionality in the TG (159). This peculiarity in the differential functionality of CD8 T-cell specificities in mice has made it somewhat difficult to understand the importance of a diverse T-cell repertoire during latent infection. Furthermore, this illustrates that there must be a refinement in the understanding of where exactly different specificities of CD8 T-cells localized within TG, whether: 1) single specificities localize around the same neuron; 2) multiple specificities localize around the same neuron; or 3) different specificities remain in different parts of the TG.

Conventionally, CD8 T-cells are cytolytic and kill virus-infected cells. Cytolytic effector function is effective in tissues that readily regenerate because this process efficiently eliminates cells destined to die. In both mouse and human TG, neuron-interacting CD8 T-cells express cytolytic factors like granzymes and perforin, yet the neurons encountered do not show signs of degeneration suggesting a neuron protective mechanism involving a tripartite role of neurons, SGC and T-cells. Human SGC express immune-inhibitory molecules [e.g. HLA-E and programmed death (PD)-1 ligand; PD-L1] that potentially restrict the cytotoxicity of T-cells (e.g. CD94/NKG2A complex and PD-1, respectively) towards neurons (133). The non-cytolytic mechanisms of CD8 T-cells to limit viral spread has been shown in mouse TG. One mechanism comes in the form of IFNγ secretion, which activates machinery in the host neuron to limit viral replication within infected neurons (160). In parallel, CD8 T-cells also release granzyme B (GrzB), which conventionally cleaves proteins that induces host cell apoptosis. In the context of HSV-1 infection, rather than inducing apoptosis, GrzB cleaves the essential immediate early protein, ICP4, which is critical for HSV-1 to exit latency. Therefore, through this mechanism GrzB can prevent HSV-1 reactivation. Currently, it is unknown why GrzB cleaves ICP4 but not the normal machinery that leads to apoptosis in this circumstance. However, one could posit that ICP4 acts as a “GrzB sink”, which would limit the levels of active GrzB. While GrzB has been predicted to cleave other viral proteins, it is unclear whether cleavage of other viral proteins occurs *in vivo*. Specifically, we know that ICP47, a protein that interrupts antigen presentation primarily in humans — less so in mice — also has GrzB cleavage sites, suggesting that GrzB may act to enhance the host cell’s ability to present antigen to T-cells (128). There is a possibility that other granzymes (humans have five, mice have eleven) act in a similar way to GrzB as mice deficient in GrzB can still control viral latency. However, mice deficient in perforin, a pore-forming molecule that allows the entry of granzymes into target cells, fail to control viral latency (128), suggesting that T cell granule contents in some way are functionally important.

Characterization of viral latency and reactivation remain a topic for discussion. Historically, viral latency was characterized by absence of lytic viral gene expression and the continual expression of the noncoding LATs (64). However, during latency in mice, CD8 T-cells specific for HSV antigens closely localize to infected neurons and form immunological synapses that include the T-cell receptor (TCR) (145). Additionally, data from humans and mice revealed that HSV-1-specific CD8 T-cells express markers of T-cell activation like programmed death (PD)-1, T-cell immunoglobulin and mucin domain-containing protein (Tim)-3, lymphocyte activation gene (LAG) 3, and others (21, 157, 159, 161–163). CD8 T-cells upregulate these proteins after stimulation suggesting *in vivo* T-cell activation. Notably, these markers are also considered markers of functional exhaustion (164), which implies a more continuous stimulation during latency. Inhibition of these exhaustion proteins can supplement CD8 T-cell functionality *in vivo.*

Preventing viral reactivation from latency relies on a functional host immune response, and disruption of this host response through steroid use, psychological stress, ultraviolet (UV) light, and other means results in loss of control of viral latency. These stressors not only suppress the immune system, but they also correlate with a loss of control of viral latency. This comes in the form of increased viral genomic DNA within the TG and a modest production and release of infectious virus (14, 165–169). Together, these data suggest that loss of viral control is a direct effect of immunosuppression. During HSV-1 infection, the TG harbors many immune cells; however, in a majority of the studies CD8 T-cells have been shown to play the most prominent role in controlling the virus. This is because TG-resident CD8 T-cells are dramatically affected by the stressors listed above (14, 142). Additionally, *in vivo* CD8 T-cell depletion has resulted in an increased viral burden in the TG (170). Finally, CD8 T-cells can effectively limit viral reactivation in TG explant cultures, and *in vitro* depletion of these cells results in a loss of control of latency (171).

While CD8 T-cells have been shown to be critical for viral control in certain models of disease, other models suggest a more modest role for CD8 T-cells in disease (129). Specifically, depletion studies of CD8 T-cells in models of primary disease and UV light-induced reactivation does not lead to increased mortality of mice or uncontrolled viral replication within the TG (172). More recently, a study showed that circulating virus-specific antibodies augment the host’s ability to prevent viral reactivation from latency (170). In the absence of these circulating antibodies, CD8 T-cells are critical to prevent viral reactivation and disease, suggesting redundant layers of immune control. A complicating factor among studies that investigate CD8 T-cell responses is the inconsistent use of virus strains across labs, methods of infection, infectious doses, and the types of measurements of viral reactivation. These inconsistencies call for a more uniform approach, including use of sequence validated low passage reference HSV-1 strains, towards critically assessing the role of immune cells including CD8 T-cells during viral latency, reactivation and disease.

The role of CD4 T-cells in HSV-1 infected mice is primarily investigated at the cornea, which is the site of sight-threatening HSK. Indeed, CD4 T-cells play important roles in corneal pathogenesis as they produce cytokines that disrupt the nerve and collagen network in the cornea. Moreover, depletion of CD4 T-cells alleviates disease at the cornea (173–175). During infection, CD4 T-cells also infiltrate the TG, but their role in TG remains enigmatic. The epitopes recognized by murine CD4 T-cells are still incompletely understood, but human TG infiltrating CD4 T-cells recognize several viral proteins including ICP47 and VP16 (138). Within murine TG, CD4 T-cells have been linked to assisting the functionality of CD8 T-cells during priming presumably through the production of inflammatory cytokines like IFNγ, which helps the establishment of viral latency during primary HSV-1 infection (176). As the infection enters the latency phase, CD4 T-cells become less inflammatory and begin to produce IL-10, which downregulates CD8 T-cell proliferation but not their effector function. In mouse models, IL-10 was detected directly *ex vivo* in TG without any additional stimulation (177). These data together with human studies that showed CD4 and CD8 T-cell conglomerates in close approximation to latently HSV-1-infected neuronal somata suggest that, similar to CD8 T-cells, CD4 T-cells continually or periodically recognize and respond to antigenic stimulation throughout latency. Because neurons lack MHC-II, infiltrating macrophages and/or more likely the ganglion-resident SGC are the local antigen presenting cell (APC) for CD4 T-cells. Human TG-resident SGC are related to macrophages and myeloid dendritic cells with regards to their phagocytic capacity and expression of CD45, co-stimulatory molecules (CD54) and both MHC-I and -II. Given their localization and phenotype, SGC are candidate APC to create an immunocompetent but not overtly inflammatory environment to support HSV-specific CD4 and CD8 T-cell responses within latently HSV-1- infected TG. Unlike CD8 T-cells, CD4 T-cells do not appear to play a prominent role in the maintenance of latency as depletion of these cells *in vivo* does not affect viral burden and only modestly affects CD8 T-cell effector function. That being said, a dual depletion of CD4 and CD8 T-cells results in a loss of viral control suggesting that CD4 T-cells are sufficient to prevent viral reactivation from latency, but may not be absolutely necessary (178).

The role of antibody-producing B-cells in maintaining viral latency is less well-defined. Both mice and humans lack secondary or tertiary lymphoid tissue in ganglia and B-cells have not been detected within the tissue in both species. Also, primary HSV-1 infection leads to a priming and expansion of antibody producing B-cells which then begin to secrete antibodies. B-cell deficiency during early infection can lead to viral dissemination to the brain, encephalitis and mortality (179, 180). Serum antibodies are effective at controlling early viral replication of highly pathogenic HSV-1 strains like McKrae or SC16 (167). While serum antibodies have not been tested in less pathogenic models of disease, recent studies have shown how maternal antibodies prevent disease and morbidity in neonatal HSV infections (181, 182), which illustrates a potential role that circulating antibodies play in the prevention of disseminated disease.

## Future Directions for Therapeutic Intervention

HSV-1 causes lifelong recurrent infections and there is currently no cure or licensed vaccine that prevents human disease. In most immunocompetent individuals, HSV-1 is generally associated with mild recurring sores in the skin and the oral or genital mucosa. However, HSV-1 causes serious diseases given the opportunity, especially in neonates and immunocompromised individuals where severe systemic diseases can develop that are often fatal. Furthermore, HSV-1 is also a major cause of infectious blindness and encephalitis worldwide. Most HSV-1-induced diseases are a consequence of reactivation of latent virus rather than primary infections. The severity of herpetic diseases appears to be associated with the level of latent and reactivatable HSV in ganglia and the efficacy of the local immune responses in both the periphery and ganglia (183–185). While the ideal anti-HSV therapy should target the latent HSV-1 reservoir in ganglia for reduction or removal, this is not yet achievable at efficient levels. As such, establishing or bolstering of the local anti-HSV immune responses in naïve and HSV-1-infected individuals, respectively, is the most appropriate strategy.

That being said, while therapy is good, prevention is better. The field of vaccines has been recently reviewed (186). Prevention of acquisition *via* anogenital and ocular routes by sex education and hygiene is a must, but this will never eliminate HSV-1 transmission as episodes of infectious HSV-1 shedding are frequent and often asymptomatic, especially in the oral cavity (7). But how good are the current therapies to treat HSV-1 infections? Antivirals, including (val) acyclovir and famciclovir, are comparatively safe and effective medications available to reduce the severity and frequency of clinical symptoms, but does not eliminate latent HSV-1, and if therapy is stopped, virus shedding and symptoms often recur (187). Furthermore, resistant HSV-1 can develop during long-term treatment in immunocompromised individuals and patients with recrudescent herpetic eye diseases (188, 189). As such, The World Health Organization has made new treatments and vaccine candidates against both HSV-1 and HSV-2, a priority (190).

Elimination of latent HSV-1 is the best option, as it would remove the source of recurrent virus that cause disease. This is particularly important for HSK, the leading cause of infectious corneal blindness in westernized societies (116). There has been considerable development in HSV genome targeting using CRISPR/Cas9 and meganuclease type strategies to reduce virus load and lower reactivation (191). Some progress has been made using gene editors delivered *via* adeno-associated virus (AAV) vectors, but the levels of gene editing were modest (192). Recently, the same group has improved this technology by including meganucleases targeting HSV-1 UL19 and UL30, delivered by three AAV serotypes (AAV1, AAV8 and AAVRh10). Treatment of latently HSV-1 infected mice, infected *via* the cornea, led to an unprecedented reduction of both latent viral genomes in ganglia and reactivating virus upon ganglion explant (125). The data are very promising, but several hurdles need to be taken to move this approach towards clinical application. Confirmation of the absence of off-target cleavage of the human genome is warranted. The species-specificity of which neuron types are latently infected, and which subsets can reactivate, is another important issue as these determine the AAV serotypes best used to deliver gene editors like meganucleases. Compared to mice (125, 193), data on human ganglionic neuron subsets in general and particularly those susceptible to HSV-1 latency and reactivation are limited (194, 195). Finally, it is preferred that the activity of gene editors is transient as constitutive expression may have deleterious effects on neuronal function, and that the delivery is targeted to specific neurons that cause recrudescent disease. In cases of HSK, this might involve vector application to the cornea for selective delivery to neurons located in the ophthalmic branch of the TG (145, 196). Data on the detailed biology of HSV-1 infected neurons in the human TG will also be of benefit in the development of strategies to strengthen immune control of latent HSV-1 in ganglia. For example, it may be possible to improve intrinsic immunity of neurons and antiviral activities of SGC using vector delivered gene augmentation in a safe and cell type-dependent manner (197, 198).

Because immunity is more manipulatable (through use of a vaccines for example), this review discusses current knowledge on intrinsic, innate and adaptive immunity involved in controlling HSV-1 infection, specifically focusing on their roles and interplay in ganglia that prevent HSV-1 reactivation. At the outset, it is clear that while immunity provides protection and control of latency, it is not complete. Sterilizing immunity to HSV-1 will be very difficult if not impossible to achieve because the virus is genetically well adapted to remain latent and produce infectious virus throughout life in many individuals with a fully intact intrinsic, innate and adaptive immunity. Furthermore, because HSV-1 gains access to neurons rapidly before the adaptive immunity is fully formed, it will be difficult to prevent latency establishment. At best, therapies should induce an efficacious and especially long-term immunity in the periphery at the sites of entry and egress, but particularly at the source of the clinical problem: latent HSV-1 in human ganglia.

Areas of future interest and study include the correlation of specific intrinsic and innate factors that contribute to the virus’ latent state in neurons. A majority of the intrinsic factors so far identified have been largely characterized in fibroblasts under conditions in which viral lytic gene expression is halted by use of antivirals or by deletion of IE genes, particularly the gene encoding ICP0; or through genetic analyses of people who are particularly susceptible to HSV reactivated disease. Some factors such as PML have been shown to have roles in human cultured neuron systems, and it will be of great interest to address how the multiple factors identified in fibroblasts apply to specific neuron subtypes that harbor latent HSV-1. Current data on innate immune control of latent HSV-1, which to some extend overlap with intrinsic immunity, hint at the potential protective role of the neuron-interacting SGC, which phenotypically and functionally closely resemble microglia in the brain. Their role as local APC of T-cells in ganglia and/or their direct antiviral role towards neurons is of interest of future studies. In comparison, the importance of CD8 T-cells, most likely CD8 T_RM_ cells, to prevent HSV-1 reactivation by different mechanisms is generally accepted and supported by data of many labs in rodent models and human post-mortem ganglia specimens. Latently HSV-1-infected ganglia also contain CD4 cells, commonly embedded in cell clusters with CD8 T-cells in close vicinity to neuronal somata (130, 131). Their fine specificity and role is still unclear: indirect role(s) by supporting CD8 T-cell function, active involvement in controlling latency, or both?

## Conclusions

To dissect the role of the three types of immunity controlling HSV-1 latency several tools and methods are now available. The experimental HSV-1 rodent models have been a boon to dissecting these, and the development of human neuron models is allowing the knowledge learned from mice to be applied to humans. There has been a particularly strong development in engineering various *in vitro* neuron models and advanced single cell approaches. However, all models and methods have their limitations as neither of them cover the full complexity and validity of their interactions in ganglia within and between species. For example, it is well established that data obtained in rodent models do not fully recapitulate the aspects of latency and reactivation of HSV-1 in its natural host. There is also the disparate data that have been obtained using different virus/mouse strain combinations, infection routes, etc. Human cadaveric ganglia specimens are of interest but they only provide a snapshot of the host/virus interplay in time. The newer *in vitro* neuron models provided detailed insight into the events occurring during the early phase of reactivation but lack the divergence of neurons seen in human ganglia, the presence of inflammatory cells and ganglion-resident cell types such as SGC. Finally the advanced single cell approaches available to study the phenotype and function of multiple cells in latently HSV-1-infected ganglia or neuron cultures in detail is of great value, but likewise human ganglion specimens only provides a snapshot of the virus/host interaction in time. Recognizing the limitations and advantages of these models and tools is a prerequisite to improve our knowledge on immune control of HSV-1 latency. Specific questions related to either/or combinations of the 3 types of immune system responses can be addressed in *in vitro* neuron model systems, including co-culture with SGC and/or T-cells, subsequently tested in the appropriate *in vivo* rodent models and validated in human ganglia specimens *in situ* or *ex vivo*. Vice versa, data obtained by advanced single cell approaches, including single cell RNA sequencing, of latently HSV-1-infected human and mouse ganglia can be assayed for functional relevance in *in vitro* neuron model system.

While most labs focus on their highly sophisticated model systems and tools, the field would benefit from integration of expertise in joint efforts aimed to tackle the global burden of HSV-1 disease. Technologies such as animal models, *in vitro* neuron model systems, HSV-1 mutant creation and validation, single cell analyses of dissociated ganglia parenchymal cells and leukocytes, epigenetic analyses of HSV-1 genomes, and imaging of excised tissues, to name a few, are specialized, resource intensive and likely beyond the reach of single research groups. The authors collaborate in the HSV-1 field and fully acknowledge this statement. Several of the outstanding questions, listed in **Table 1**, are currently under investigation by members of this consortium.

**Table 1:** Outstanding questions of HSV latency, reactivation and pathogenesis.

Does HSV-1 reactivation lead to death of infected neurons?
What differentiates HSV from host cell DNA during innate DNA sensing?
Can cell-intrinsic antiviral activities in ganglia be augmented, for example by promoting repressive histone modifications?
What are the role(s) of satellite glial cells in immune containment of latent HSV-1?
Are virus-specific T-cells in ganglia reactive epiphenomena or active participants in modulating HSV-1 reactivations? Can adaptive HSV-1 immunity be be augmented to reduce clinical shedding/transmission thresholds?
What factor(s) govern immunodominance and development of the HSV-1-specific CD8 and CD4 T-cell repertoire in ganglia?
What is the transcriptional profile and fine specificity of HSV-1-specific T cells in human ganglia?
What specific subtypes of neurons are latently infected with HSV-1 in humans and what subtype-specific factors predispose neurons to reactivation?
What viral and lumbosacral ganglionic factors and mechanisms determine the milder clinical course and shedding frequency of recurrent genital HSV-1 compared to HSV-2?
What role(s) does the local and/or systemic microbiome play in HSV-1 infections?

## Author Contributions

All authors contributed to the article and approved the submitted version.

## Funding

GV is supported by the National Institute of Allergy And Infectious Diseases of the United States National Institutes of Health under Award Numbers AI151290, AG064800-02S2 and 75N93019C00063. DMK is supported by AG064800-02S2 and 75N93019C00063. PK is supported by grants from the National Institutes of Health under award numbers AI151290, EY015291, NS064022 and AI122640. AS is supported by grants from the National Institutes of Health under award numbers EY032482, EY025761, and EY026891. AS and PK also acknowledge support from a P30 CORE grant (EY08098), and unrestricted support awards from Research to Prevent Blindness Inc, NY and The Eye & Ear foundation of Pittsburgh.

## Conflict of Interest

DK receives research funding from Sanofi Pasteur related to HSV vaccines and is co-inventor of institutionally-owned patents concerning HSV vaccines.

The remaining authors declare that the research was conducted in the absence of any commercial or financial relationships that could be construed as a potential conflict of interest.

## Publisher’s Note

All claims expressed in this article are solely those of the authors and do not necessarily represent those of their affiliated organizations, or those of the publisher, the editors and the reviewers. Any product that may be evaluated in this article, or claim that may be made by its manufacturer, is not guaranteed or endorsed by the publisher.
